# Deacetylation of HSC70-4 Promotes *Bombyx mori* Nucleopolyhedrovirus Proliferation via Proteasome-Mediated Nuclear Import

**DOI:** 10.3389/fphys.2021.609674

**Published:** 2021-02-19

**Authors:** Fuxiang Mao, Xi Chen, Jonas Ngowo, Yajie Zhu, Jihai Lei, Xu Gao, Meng Miao, Yanping Quan, Wei Yu

**Affiliations:** ^1^Institute of Biochemistry, College of Life Sciences and Medicine, Zhejiang Sci-Tech University, Hangzhou, China; ^2^Zhejiang Provincial Key Laboratory of Silkworm Bioreactor and Biomedicine, Hangzhou, China

**Keywords:** HSC70-4, BmNPV, deacetylation, nuclear import, proteasome

## Abstract

Silkworm (*Bombyx mori*) is a model organism with great agricultural economic value that plays a crucial role in biological studies. *B. mori* nucleopolyhedrovirus (BmNPV) is a major viral pathogen found in silkworms, which leads to huge silk loss annually. In a recent lysine acetylome of silkworm infected with BmNPV, we focused on the heat shock cognate protein 70-4 (HSC70-4) lysine acetylation change due to the consequent nuclear accumulation and viral structure assembly. In this study, the genome replication, proliferation, and production of budded viruses (BVs) were arrested by HSP/HSC70 inhibitor treatment. However, HSC70-4 overexpression enhanced BmNPV reproduction. Furthermore, site-direct mutagenesis for acetylated mimic (K/Q) or deacetylated mimic (K/R) mutants of HSC70-4 demonstrated that lysine 77 (K77) deacetylation promotes HSC70-4 stability, viral DNA duplication, and HSC70-4 nuclear entry upon BmNPV challenge, and the nuclear propulsion of HSC70-4 after viral stimulus might be dependent on the interaction with the carboxyl terminus of HSC70-interacting protein (CHIP, an E3 ubiquitin ligase), followed by ubiquitin-proteasome system assistance. In this study, single lysine 77 deacetylation of HSC70-4 was deemed a part of the locomotive pathway for facilitating BmNPV proliferation and provided novel insights into the antiviral strategic development.

## Introduction

Silkworms play an essential role in the ancient Silk Road trade because of their derivative silk with high tremendous economic value, but are also of significance in research with respect to ease of rearing, acquisition of genome sequence, and availability of mutants from genetically homogeneous inbred lines (Xia et al., 2004). *Bombyx mori* nucleopolyhedrovirus (BmNPV), the primary pathogenic agent in silkworm viral disease, includes a large circular double-stranded DNA genome with putative 143 open reading frames (Shen et al., 2018). In addition, two distinct virion phenotypes are responsible for disseminating in insects or cells, respectively (Jiang and Xia, 2014). One is the occlusion-derived virus (ODV), which contains numerous virions within a crystallized protein, called polyhedron, that promotes oral infection. The other is the budded virus (BV) that spreads between internal tissues. A detailed baculovirus invasion mechanism and silkworm immune response still need further understanding (Jiang et al., 2021a).

Heat shock proteins are involved in the interaction between baculovirus and silkworms (Mao et al., 2020; Shang et al., 2020; Jiang et al., 2021b). Heat shock protein 70 (HSP70) is conserved across evolution from archaebacteria to higher mammals (Lindquist and Craig, 1988). Differing from the HSP70 response to stress condition, heat shock cognate protein 70 (HSC70) is constitutively expressed to maintain the protein folding under normal conditions (Gething and Sambrook, 1992). Several investigations recently indicated that baculovirus infection induces *HSP/HSC70s* expression to promote viral genome replication, protein synthesis, and BV production (Lyupina et al., 2010, 2011, 2013, 2010; Breitenbach and Popham, 2013). For BmNPV and silkworm, HSC70 was found in the protein composition of ODV virion (Liu et al., 2008), and the transcriptional activity of the HSC70-4 promoter was elevated by the BmNPV homologous region 3 (Tang et al., 2005). In addition, HSC70-4 was accumulated in the nucleus at a very late BmNPV infection phase and identified the embedded assembly in ODV and BV structure, including the envelope and capsid (Iwanaga et al., 2014). During the polyhedrin aggregates/aggresomes formation upon BmNPV infection, HSP/HSC70s and ubiquitinated proteins colocalized with polyhedrin aggregates/aggresomes (Guo et al., 2015). Moreover, HSC70-4 interplays with the E3 ubiquitin ligase, carboxyl terminus of HSC70-interacting protein (CHIP), in *B. mori* (Ohsawa et al., 2016). Interestingly, BV production and polyhedrin expression of BmNPV is dependent on the intact ubiquitin-proteasome system (Katsuma et al., 2011). Based on the above reports, although HSC70-4 plays a crucial role in BmNPV infection, the elaborate molecular mechanisms need to be elucidated further.

Post-translational modifications, such as acetylation (Mawatari et al., 2015), phosphorylation (Muller et al., 2013), methylation (Gao et al., 2015), and ubiquitination (Kundrat and Regan, 2010), are essential for flexible regulation of HSP/HSC70s functional alternatives. Acetylation, which used to be studied in histone proteins, is also a commonly reversible molecule switch for non-histone proteins, affecting many cellular processes (Verdin and Ott, 2015). Currently, HSP/HSC70 acetylation has been widely studied in many aspects of cellular homeostasis, which is associated with protein folding, degradation, apoptosis, and autophagy (Yang et al., 2013; Wu et al., 2014; Seo et al., 2016; Park et al., 2017; Sun et al., 2019). For example, in the early stress period, the acetylated K77 lysine site of HSP70 led to increased protein refolding via interaction with HSP70/90 organizing protein (HOP) and HSP90; however, in the late stimulus phase, deacetylated K77 contributed to protein degradation by association with CHIP and HSP40 (Seo et al., 2016). In addition, K77 acetylation also hinders the caspase-dependent/independent apoptosis via interplay with Apaf1/AIF, respectively (Park et al., 2017). Similarly, HSP/HSC70s K88, K126, K159, and K246 acetylation-mediated protein-protein interaction, apoptosis, and autophagy have been widely investigated in cancer cells (Yang et al., 2013; Wu et al., 2014; Sun et al., 2019). Our previous proteomic profiling presented that BmN cellular histone deacetylase (HDAC) was upregulated upon BmNPV challenge (Mao et al., 2018). Nowadays, due to the analogous hydrophobic property, glutamine (Q) and arginine (R) are typically used for mimicking lysine (K) acetylation and deacetylation, respectively (Fujimoto et al., 2012; Huang et al., 2015). Nonetheless, how the HSP/HSC70s acetylation modulates viral proliferation is yet unknown.

Silkworm protein acetylation was studied in pro-survival, apoptosis, and autophagy (Zhou et al., 2016; Xue et al., 2019; Yang et al., 2020). Our recent acetylome upon BmNPV infection also stimulated a focus on HSC70-4 acetylation performance in baculovirus replication (Hu et al., 2018). In this study, we used the HSP/HSC70 inhibitor or overexpression of HSC70-4 to determine viral genome replication, propagation, and BV release. Furthermore, we detected several lysine sites by acetylation-mimic (K/Q) or deacetylation-mimic (K/R) in viral DNA duplication, and K77 deacetylation of HSC70-4 increased the number of viral genome copies by enhanced stability and nuclear import that may be dependent on the interaction with CHIP, followed by the ubiquitin-proteasome system for propulsion. This finding unveils the baculovirus-host interaction mechanism and provides novel insights into the antiviral strategy development.

## Materials and Methods

### Plasmids, Cells, and Viruses

*Bombyx mori* BmN cell line, originated from the silkworm ovarian tissue, was preserved at 27°C in Sf-900 medium (Thermo Fisher Scientific, United States) supplemented with 10% fetal bovine serum (FBS; Corning, United States). BmNPV and the enhanced green fluorescent protein (EGFP)-tagged virus (BmNPV-EGFP), harboring the EGFP under the polyhedrin promoter without any protein fusion, were sustained in our laboratory with the multiplicity of infection (MOI) 10 for differently treated cells. The recombinant plasmid pET28a-*HSC70-4*(898-1801) for the induction of target protein expression and purification was constructed as described previously (Iwanaga et al., 2014). The transient expression vector in eukaryotic BmN cells with pIEx-1-*HSC70-4* was achieved for overexpression studies, and the target genes *HSC70-4*, *CHIP*, and *HOP* were amplified from the BmN cells. For this, RNA was isolated from BmN cells using TRIzol reagent (Thermo Fisher Scientific, United States), and the cDNA was reverse-transcribed by RevertAid First Strand cDNA Synthesis Kit (Thermo Fisher Scientific). Site-directed mutagenesis in HSC70-4 (K71Q, K71R, K77Q, K77R, K88Q, K88R, K126Q, K126R, K246Q, K246R, K524Q, and K524R) was carried out by overlapping polymerase chain reaction (PCR), as described previously (Ho et al., 1989). The method was also applied for fusing EGFP with HSC70-4 (wild-type, K77Q, K77R) followed by insertion into the pIEx-1 vector. Pairs of yeast two-hybrid plasmid pGBKT7-*HSC70-4/K77Q/K77R* and pGADT7-*CHIP/HOP* were constructed to test the protein-protein interaction. All primers are listed in Supplementary Table 1.

### Antibodies, Reagents, and Transfection

pET28a-*HSC70-4*(898-1801) plasmid was transformed into *E. coli* (BL21 DE3) competent cells for recombinant HSC70-4 expression, induced by isopropyl-β-D-thiogalactopyranoside (IPTG), followed by the Ni-NTA column (Qiagen, Germany) purification. Subsequently, the refined protein was utilized for immunizing rabbits to obtain polyclonal antibodies (HuaAn Biotechnology, China). Gp64, His-tagged (Santa Cruz Biotechnology, United States), β-tubulin, and horseradish peroxidase (HRP)-conjugated secondary antibodies (Biosharp Life Sciences, China) were employed. VER155008 (VER) and MG132 were purchased from MedChemExpress (United States) and solubilized in dimethyl sulfoxide (DMSO) for the stock concentration of 50 mM. Transfection was performed as described previously (Xue et al., 2019) using SuperFectin^TM^II *in vitro* DNA Transfection Reagent (Shanghai Pufei Biotechnology, China).

### Chemicals Treatment and MTT Assay

After the chemical treatment with 1, 5, 10, and 20 μM VER for 24 or 48 h, the cells were harvested for cell viability assay. The MTT assay was performed as described previously (Yu et al., 2013b). Then, BmN cells were treated with 5 μM MG132 to bypass the cytotoxicity, based on the previous study (Katsuma et al., 2011).

### Western Blotting

Disparate treated, transfected, or infected samples were collected and lysed for extraction of total protein in cell lysis buffer containing 0.5% NP40, 150 mM NaCl, 1 mM ethylenediaminetetraacetic acid (EDTA), 50 mM Tris pH 7.5, and protease inhibitor cocktail (Bimake, United States). After 30 min lysis on ice, the whole protein extract was subjected to centrifugation at 12000 rpm, 4°C for 15 min. The protein samples were quantified by Bradford assay, and an equivalent of 20 μg was resolved by 12% sodium dodecyl sulfate-polyacrylamide gel electrophoresis (SDS-PAGE), followed by electroblotting on polyvinyl difluoride (PVDF) membranes. Then, the membranes were blocked with 5% skim milk for 2 h and probed with the corresponding primary antibody. Subsequently, the membrane was incubated with a secondary antibody, and the immunoreactive bands were visualized using SignalFire ECL Reagent (Cell Signaling Technology, United States).

### Viral Titer Determination

The viral titer of all the different groups was measured by the 50% tissue culture infective dose (TCID_50_) of BmN cells. First, the cells were transfected with empty vector pIEx-1 or pIEx-1-*HSC70-4* or treated by VER (10 μM) or DMSO, respectively, and then infected with BmNPV at an MOI of 10 for 72 h. Subsequently, the virus in the supernatant was harvested and serially diluted 10-fold from 10^–1^ to 10^–8^. A volume of 100 μL of the different gradient virus was inoculated into 96-well plates, and the titer was recorded at 0, 24, 48, 72, and 96 h p.i. by TCID_50_ endpoint dilution assay.

### Quantitative Analysis of Viral DNA Synthesis

qPCR was used to analyze viral DNA duplication as described previously (Yu et al., 2013a; Zhao et al., 2016). *gp41*, the viral gene, was applied to quantify viral DNA load and the specific primers used in qPCR to amplify the corresponding product. The qPCR was carried out using a GoTaq qPCR Master Mix kit (Promega, United States) on an ABI Prism 7500 Sequence Detection System (Applied Biosystems, United States). The PCR procedure was as follows: pre-denaturation at 95°C for 10 min, followed by 40 cycles of denaturation at 95°C for 10 s, annealing at 50°C for 10 s, and elongation at 72°C for 12 s. Each assay was carried out in biological triplicates.

### Fluorescence Microscopy

*Bombyx mori* nucleopolyhedrovirus-EGFP was used for the determination of viral propagation under differentially transfected/treated BmN cells and were observed using an inverted fluorescence microscope (Eclipse, TE2000-U, Nikon, Japan). The EGFP-HSC70-4/K77Q/K77R subcellular nucleocytoplasmic distribution upon BmNPV infection was detected under a confocal microscope (IX81-FV1000, Olympus, Japan).

### Yeast Two-Hybrid Assay

Recombinant pGBKT7-HSC70-4/K77Q/K77R and pGADT7-HOP/CHIP (2.5 μg each) constructs were simultaneously co-transformed into Y2HGold yeast competent cells AH109. The transformed yeasts (100 μL) were plated on SD-Trp/-Leu/-His/-Ade/X-α-gal nutrient-deficient medium for 3–5 days. A single colony of blue yeast was picked for another round of color observation.

### Statistical Analysis

All experiments were independently repeated at least three times, and the data are shown as means ± standard deviation. The cell viability, viral DNA amount, and viral titer were determined using Student’s *t*-test and GraphPad Prism 7. ^∗^*p* < 0.05 indicates a statistically significant difference.

## Results

### Impaired ATPase Activity of HSP70/HSC70 Interferes With Viral Proliferation

**FIGURE 1:**
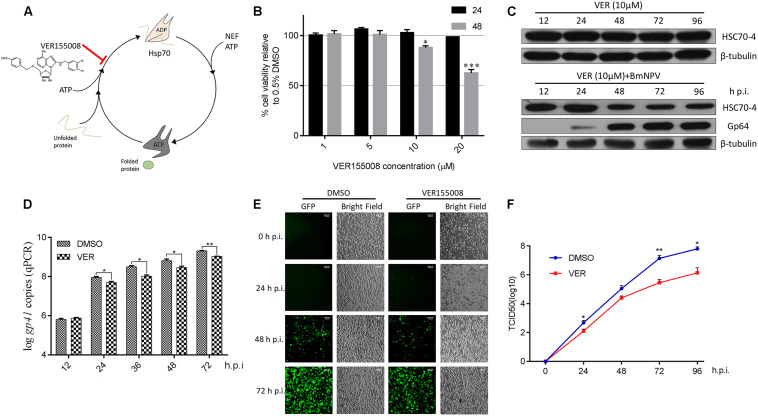
The effect of HSP/HSC70s inhibitor VER for viral proliferation. **(A)** The schematic illustration of HSP/HSC70s inhibitor VER effect on intact nucleotides’ binding cycle. **(B)** Determination of BmN cell viability by MTT assay with different concentrations (1, 5, 10, and 20 μM) inhibitor VER or DMSO incubation for 24 or 48 h in the absence of BmNPV. Cell viability is presented relative to the data of 0.5% DMSO. **(C)** Endogenous stability of HSC70-4 after inhibitor (10 μM) was added at 0 h post-infection (p.i.) or combined with inhibitor (10 μM) at 0 h p.i. and BmNPV treatment at several different time points and assessed by immunoblotting analysis. The viral structure protein Gp64 represented the BmNPV infectious process successfully. β-tubulin served as the loading control. **(D)** qPCR analysis of viral genome copies by HSP/HSC70s inhibition (10 μM) at 0 h p.i. in distinctive viral phases. DMSO groups were assessed as a negative control. **(E)** VER or DMSO-treated BmN cells were incubated with BmNPV-EGFP (enhanced green fluorescent protein). Infected cells (EGFP-positive) were detected at 0, 24, 48, and 72 h p.i. by fluorescence microscopy. Scale bar was 100 μm. The bright field represented the cell numbers and state control. **(F)** The yield of infectious BVs in the supernatants of corresponding treated cells was measured by TCID_50_ endpoint dilution assay. Each data point represented the average titer of independent biological triplicates. *p < 0.05 indicated significant difference and **p < 0.01, ***p < 0.005 indicated extreme significant difference.

Based on previous studies about ATP-mimic molecule HSP/HSC70 specific inhibitor VER (Figure 1A) suppressing flavivirus (Taguwa et al., 2015, 2019), nairovirus (Surtees et al., 2016), and baculovirus (Lyupina et al., 2014; Mao et al., 2020), we also applied this inhibitor for determining the HSP/HSC70 ATPase activity for BmNPV reproduction. Initially, the BmN cell viability after VER treatment was measured (Figure 1B), showing that the different doses of chemicals had no cytotoxicity at the early stage of 24 h, but 10 μM inhibitor reduced cell survival after 48 h. However, during the BmNPV infectious phases, host cell viability was mainly governed by the virus rather than the inhibitor. Thus, considering relevant investigations about VER-treated Sf9 cells and AcMNPV (Lyupina et al., 2014), we consequently selected 10 μM for subsequent viral trials. We also assessed whether the stability of HSC70-4 was affected when the ATPase activity was blocked or combined with viral disruption. Consequently, the protein level did not show any obvious change by chemical treatment, but a gradual decline after simultaneous virus and HSP/HSC70 inhibitor stimulus was noted (Figure 1C). Next, we incubated the virus with VER or DMSO-treated BmN cells, and the different infectious stages were collected. The findings were consistent with the total viral DNA amount (Figure 1D), BmNPV propagation (Figure 1E), and BVs production (Figure 1F) that declines after HSP/HSC70s ATPase activity impairment with infection progress. The intact HSP/HSC70s played crucial roles in BmNPV proliferation.

### Overexpression of HSC70-4 Facilitates BmNPV Infection

**FIGURE 2:**
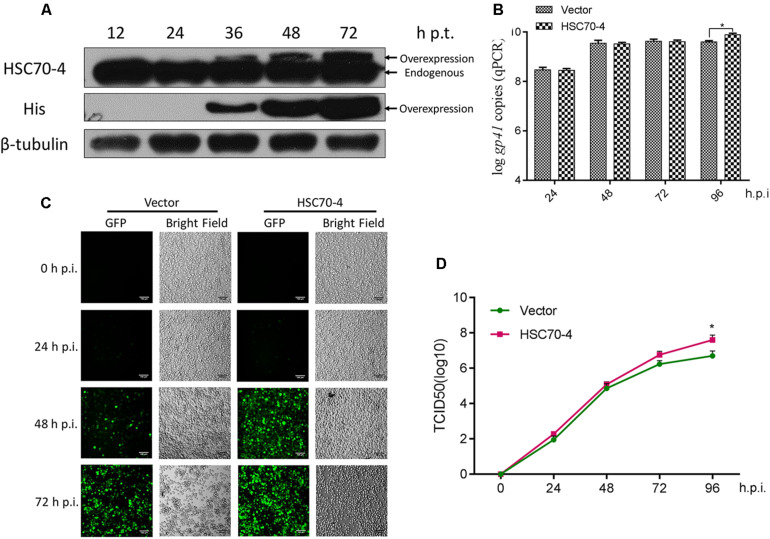
Overexpression of HSC70-4 for viral proliferation. **(A)** The transient expression level of exogenously transfected BmN cells in the absence of BmNPV challenge was measured by immunoblotting analysis. **(B)** After 48 h transfection, empty vector or HSC70-4-transfected cells were incubated with BmNPV for 24, 48, 72, and 96 h infection. The viral genome replication of the corresponding treated cells was analyzed by qPCR. **(C)** At 48 h post-transfection of pIEx-1 or pIEx-1-HSC70-4 in BmN cells, BmNPV-EGFP was added, and the infected cells (EGFP-positive) were evaluated at 0, 24, 48, and 72 h post-infection by fluorescent microscope. Scale bar was 100 μm. **(D)** The collected infectious BVs in the supernatants of differentially transfected cells were determined by TCID_50_ endpoint dilution assay. Each point was measured in biological triplicates. *p < 0.05 indicated a significant difference.

In this present study, the exogenous transient transfection indicated that overexpression of HSC70-4 is capable of being recognized explicitly as the endogenous cellular HSC70-4 by the customized polyclonal antibody (Figure 2A), and the overexpressed HSC70-4 reached a substantial level after 48 h post-transfection. Therefore, in the subsequent experiments, we adopted this time point for studying the overexpression of HSC70-4 effect in viral challenge. With this consequence, the viral genome replication (Figure 2B), BmNPV proliferation (Figure 2C), and BV yield (Figure 2D) were measured in empty vector or HSC70-4-transfected BmN cells, respectively. These data demonstrated that HSC70-4 enhances viral replication.

### Potential Lysine Acetylation of HSC70-4 Upon Baculovirus Challenge

**FIGURE 3:**
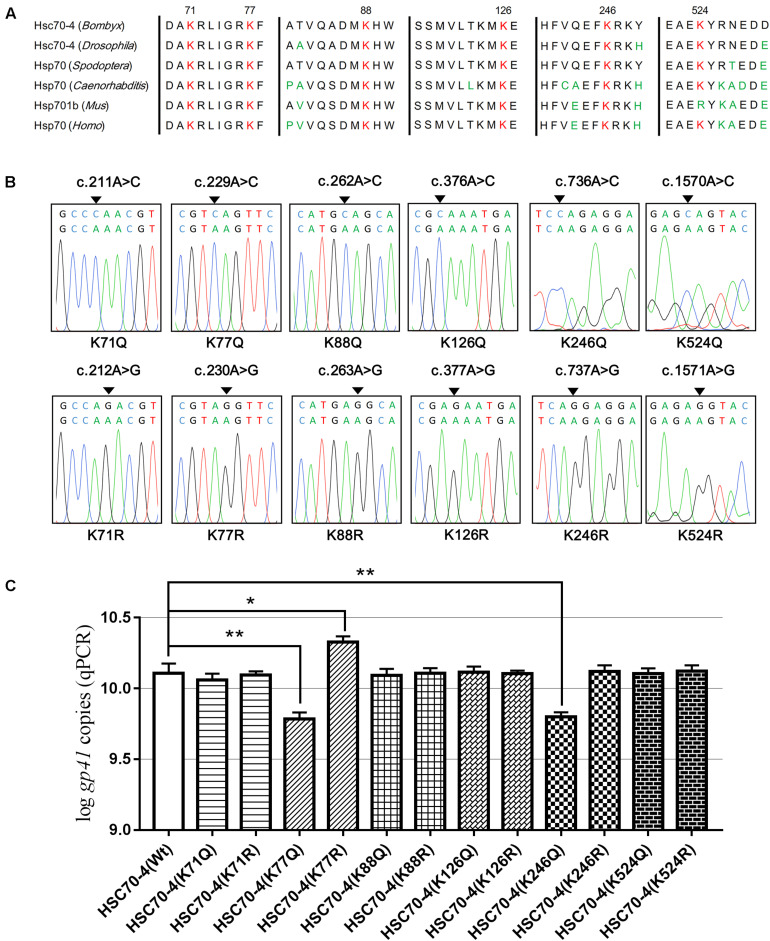
Six conserved lysine residues mutated for viral genome analysis. **(A)** K71, K77, K88, K126, K246, and K524 conserved in *B. mori* HSC70-4 were aligned with their homologs in other species among *Drosophila melanogaster*, *Spodoptera frugiperda*, *Caenorhabditis elegans*, *Mus musculus*, and *Homo sapiens*. **(B)** Sequences of HSC70-4 site-mimic acetylation/deacetylation mutants were corrected by BLAST against GenBank. **(C)** Wild-type or 12 lysine acetylated/deacetylated mimic mutants of HSC70-4 48 h post-transfection were followed by BmNPV genome replication analysis after infection for 48 h. *p < 0.05 represented a significant difference, and **p < 0.01 indicated extreme significant difference.

To study the acetylation of HSC70-4 in BmNPV, several lysine-acetylated sites (Kac) were identified in our previous relevant acetylome profiling post-BmNPV challenge (Hu et al., 2018). Also, some key Kacs, such as K71, K88, K126, K159, and K246, were investigated in recent studies (Yang et al., 2013; Wu et al., 2014; Seo et al., 2016; Park et al., 2017; Sun et al., 2019). Thus, the relatively comprehensive profile of Kac in different domains of HSC70-4 was created to represent a clear atlas (Supplementary Figure 1A). In the previous profile post-baculovirus challenge, five Kac sites were determined and analyzed by HPLC/MS/MS (Supplementary Figure 1B), while K77 and K246 in other species HSP/HSC70s had been investigated in-depth in protein folding/degradation, apoptosis, and autophagy (Wu et al., 2014; Seo et al., 2016). Related reports and the above results about HSC70-4 in BmNPV further prompted us to investigate whether the Kac response to viral stress plays functional roles in viral progress. Hence, we selected six conserved and well-studied lysine residues for further viral effects (Figure 3A). Then, overlapping PCR was employed for site-directed mutagenesis of lysine to mimic acetylation (glutamine, K/Q) or deacetylation (arginine, K/R) for viral genome replication analysis (Figure 3B). The results showed that acetylated K77 and K246 of HSC70-4 decrease the BmNPV genome copies but deacetylated K77 increases the number of copies (Figure 3C).

### K77 Deacetylation Promotes HSC70-4 Stability and Nuclear Import Upon BmNPV

**FIGURE 4:**
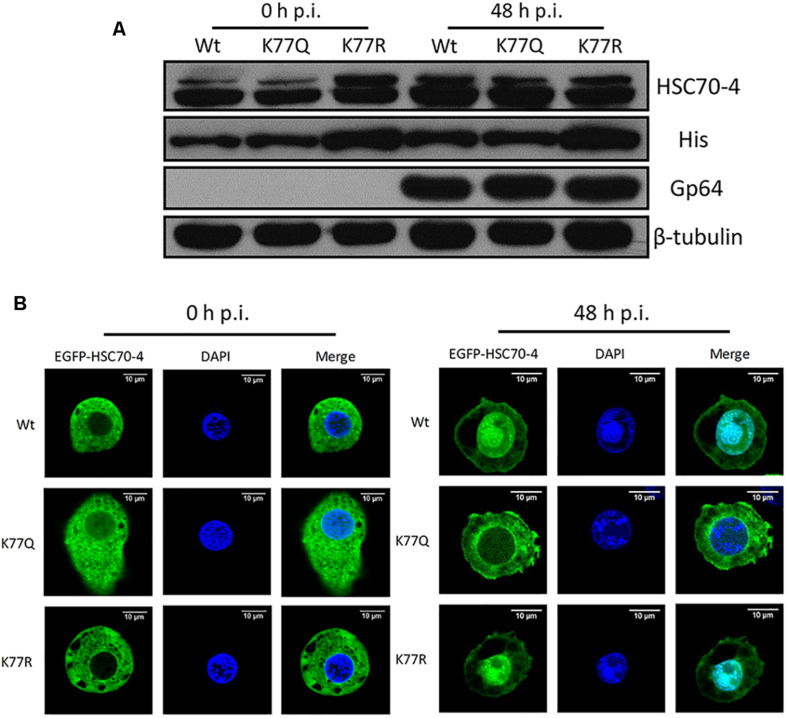
K77 residue deacetylation is vital for HSC70-4 stability and nuclear import upon BmNPV. **(A)** After 48 h post-transfection of wild-type or mutant HSC70-4, virus-treated 48 h or virus-free 48 h for determining protein level. 6× His antibody was used for detecting exogenous HSC70-4 and mutants. HSC70-4 polyclonal antibody was used for confirming the His-tagged results. Tubulin was loading control, and Gp64 represented successful infection. **(B)** Confocal microscopy was applied to analyze the subcellular localization of EGFP-tagged HSC70-4/K77Q/K77R after the BmNPV challenge. Scale bar was 10 μm. DAPI was used for nuclear indication.

In order to explore if the acetylation of K77 affected HSC70-4 stability under normal conditions or viral stress, Western blot analysis was performed to observe the protein abundance after BmNPV 48 h transfection. Results showed that deacetylated K77 was able to increase the HSC70-4 level in the presence of a virus or a virus-free situation (Figure 4A), which might contribute to enhancing viral genome copy. Based on HSC70-4 nuclear accumulation upon BmNPV (Iwanaga et al., 2014), we deduced the differential modification of this protein that would make a difference in the nuclear movement by viral propulsion. The confocal microscopy (Figure 4B) confirmed the hypothesis that the deacetylated K77 residue is valuable for HSC70-4 nuclear import under BmNPV stimulation; however, the acetylated lysine 77 site is unable to accomplish the nucleus transportation. In conclusion, the results suggested that K77 deacetylation-mediated HSC70-4 stability and nuclear import potentially facilitates BmNPV replication.

### K77 Deacetylation Is Crucial for HSC70-4 Interacting CHIP

**FIGURE 5:**
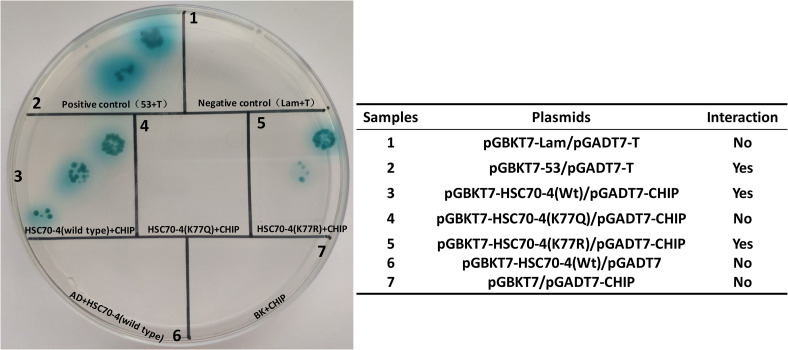
Preferential interaction between K77 deacetylation and CHIP. Yeast two-hybrid assay of the interaction between HSC70-4/K77Q/K77R and *B. mori* CHIP. pGBKT7-HSC70-4/K77Q/K77R and pGADT7-CHIP is the experimental group; pGBKT7-53 and pGADT7-T is a positive control; pGBKT7-Lam and pGADT7-T, pGBKT7 and pGADT7-CHIP, pGBKT7-HSC70-4/K77Q/K77R, and pGADT7 constitute the negative control.

A previous study reported that the K77 acetylation enhances the interplay between HSP70 and HOP, while K77 deacetylation contributes to HSP70 and CHIP interaction to implement the protein degradation (Seo et al., 2016). In *B. mori*, HSC70-4 was also capable of interacting with the E3 ubiquitin ligase CHIP (Ohsawa et al., 2016). Thus, we detected whether the K77 acetylation or deacetylation influenced the interplay between HSC70-4 and CHIP/HOP by yeast two-hybrid assay. The findings revealed that K77 acetylation or deacetylation did not cause any difference in the association between HSC70-4 and HOP in yeast two-hybrid assay (Supplementary Figure 2); however, the wild-type and deacetylation-mimic HSC70-4 still maintained the interaction with CHIP, but the acetylation-mimic K77 hindered the association with CHIP (Figure 5). Consistently, these phenomena also reached a consensus with a previous report (Seo et al., 2016). The above trials showed that this lysine 77 residue deacetylation is essential for HSC70-4 and CHIP cooperation.

### HSC70-4 Propulsion by K77 Deacetylation Requires the Ubiquitin-Proteasome System

**FIGURE 6:**
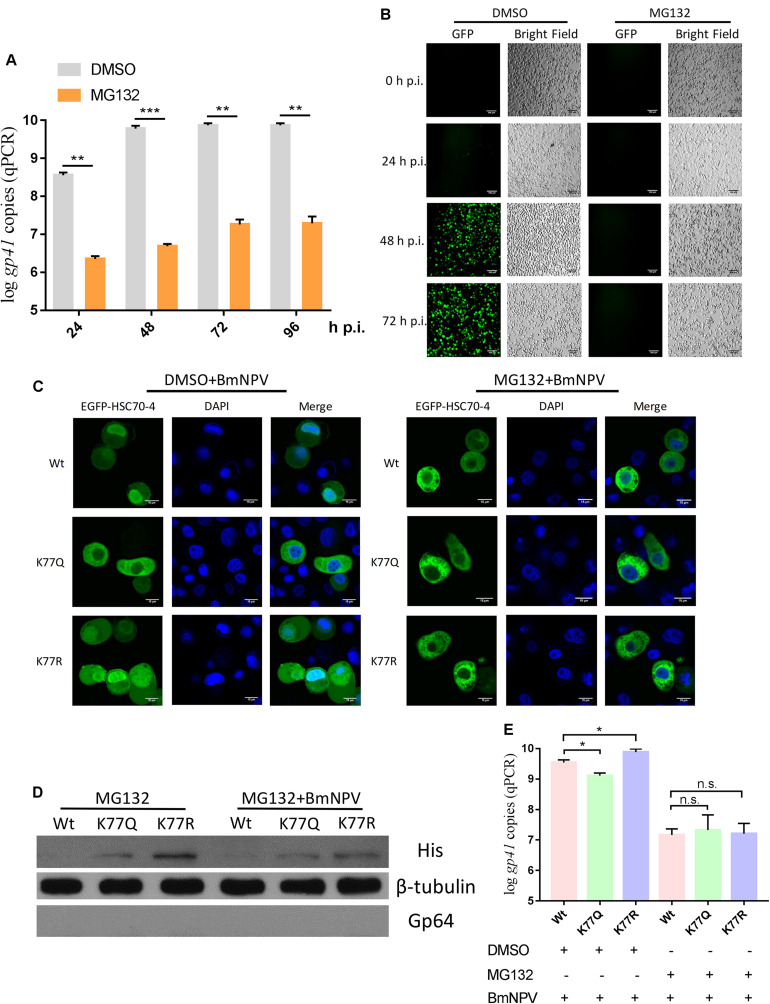
Proteasome is required for HSC70-4 nuclear accumulation and viral DNA replication. **(A)** 5 μM proteasome inhibitor MG132 (0 h p.i.) effect for BmNPV genome copies at 24, 48, 72, and 96 h p.i. DMSO was used as the normal control. **(B)** BmNPV-EGFP proliferation upon MG132 treatment after 0, 24, 48, and 72 h p.i. was recorded by a fluorescence microscope. Bright field indicated the BmN cell number and cellular state. Scale bar was 100 μm. **(C)** After 48 h transfection of EGFP-HSC70-4/K77Q/K77R, MG132/DMSO (0 h p.i.), and BmNPV treatment, BmN cells were cultured for another 48 h post-infection and observed through confocal microscopy. DAPI was used to indicate the nucleus. Scale bar was 15 μm. **(D)** MG132 (0 h p.i.) or BmNPV was added simultaneously at 48 h post-transfection of wild-type or mutant HSC70-4 for 48 h incubation, followed by Western blot analysis. 6× His antibody was used for detecting exogenous HSC70-4 and mutants. Tubulin was used as a loading control. Gp64 represented viral infection progress. **(E)** Correspondingly, HSC70-4 or mutants at 48 h post-transfection were supplemented with inhibitor and virus to the transfected cells 48 h p.i. for the analysis of viral DNA amount. n.s. means non-significant difference. *p < 0.05 represents significant difference and **p < 0.01, ***p < 0.005 indicates extremely significant difference.

The previous investigation demonstrated that the intact ubiquitin-proteasome system is crucial for BV production and polyhedrin expression during BmNPV infection (Katsuma et al., 2011). Combined with the K77 acetylation-induced difference between E3 ubiquitin ligase CHIP interaction and HSC70-4, we attempted to find if the ubiquitin-proteasome is a potential alternative pathway for HSC70-4 propulsion to the nucleus. Therefore, the application of proteasome inhibitor MG132 for analyzing BmNPV genome replication and proliferation manifested that the robust proteasome played vital roles in viral pathogenesis, such as genomic duplicates (Figure 6A) and propagation (Figure 6B), which was in agreement with the previous results (Katsuma et al., 2011). Furthermore, the damaged proteasome hampered HSC70-4 nuclear import (Figure 6C), viral protein synthesis (Figure 6D), and viral genome copies (Figure 6E) after BmNPV invasion irrespective of whether it is acetylated or deacetylated. Although HSC70-4 is essential for the substrate degradation through the ubiquitin-proteasome system (Fernández-Fernández et al., 2017), the BmNPV utilized in this pathway demands more elucidation. These consistent consequences potentially indicated that HSC70-4 nuclear accumulation upon baculovirus challenge might be modulated by ubiquitin-mediated proteasome function.

## Discussion

*Bombyx mori* nucleopolyhedrovirus (BmNPV) is a pathogen that threatens the survival of silkworms; however, the baculovirus expression vector system could be used for the commercial manufacture of protein mass. Owing to the ambiguous mechanism between BmNPV and silkworm, we pursued the molecular machinery underlying this sophisticated process. Based on our previous BmN cellular acetylome upon BmNPV infection, five lysine residues with acetylated change were identified in HSC70-4 (Hu et al., 2018). This finding stimulated us to deduce whether this posttranslational modification played regulatory roles in the pathogenesis and development of baculovirus. In our study, K77 deacetylated HSC70-4 interacted with CHIP, assisted by the proteasome to accumulate in the nucleus for facilitating BmNPV genome replication.

Firstly, in the present study, we applied a wide-spectrum HSP/HSC70 inhibitor VER to test its function for BmNPV. VER was previously used to determine the *Autographa californica* multiple nucleopolyhedrovirus (AcMNPV) viral protein synthesis, genome replication, and BV production (Lyupina et al., 2014). In agreement with this phenomenon, VER also exerted an inhibitory role in BmNPV genome replication (Figure 1D), proliferation (Figure 1E), and BV yield (Figure 1F). Different from 20 μM or 100 μM VER treatment for Sf9 cells (Lyupina et al., 2014), the moderate application of 10 μM VER in BmN cells was able to diminish the cytotoxic effect (Figure 1B). Surprisingly, HSC70-4 protein level declined upon VER and BmNPV combined treatment (Figure 1C), which could be inferred as proper functions of HSC70-4 in baculovirus propagation.

Several studies investigated HSP/HSC70 in AcMNPV-infected Sf9 cells and reported that gene expression and protein abundance of HSP/HSC70 is upregulated in infected cells (Lyupina et al., 2010, 2011, 2013). However, Iwanaga et al. (2014) reported that HSC70-4 is steady during BmNPV invasion (Liu et al., 2008), which is consistent with our confirmation (Supplementary Figure 3). Combined with the above results of BmNPV and VER treatment, it is speculated that the inhibitor-impaired HSC70-4 would be degraded after the virus challenge, which possibly meant that BmNPV could distinguish the intact or damaged HSC70-4 for further utilization. The following data also supported that HSC70-4 is beneficial for baculovirus proliferation (Figure 2C), genome replication (Figure 2B), and BV release (Figure 2D).

In light of our recent silkworm cell acetylated profiling on baculovirus infection (Hu et al., 2018), several lysine residues (K77, K100, K246, K524, and K557) were identified in HSC70-4 with dynamic acetylation triggered by BmNPV (Supplementary Figure 1B). Hence, in association with the above results and other existing HSP70 acetylation reports (Yang et al., 2013; Wu et al., 2014; Seo et al., 2016; Park et al., 2017; Sun et al., 2019), we chose six relatively conserved lysine sites (K71, K77, K88, K126, K246, and K524) to continue the exploration of HSC70-4 in the virus progression (Figure 3A). After site-specific mimic acetylation (lysine/glutamine, K/Q) or deacetylation (lysine/arginine, K/R) mutation (Figure 3B), the viral genome analysis indicated that K77 and K246 acetylation of HSC70-4 showed a compromised effect in comparison to that of wild-type HSC70-4, while K77 deacetylation of HSC70-4 had a more robust influence than that of wild-type HSC70-4 (Figure 3C). Wu et al. (2014) investigated that K246 deacetylation of HSP70 was deacetylated by HDAC1 and HDAC7 that, in turn, inhibited autophagic cell death. Seo et al. (2016) demonstrated that HSP70 with K77 acetylation was effectuated by ARD1 acetyltransferase. The protein interacted with HSP90 and HOP for refolding as a response to early stress. In the late stimulus, HSP70 with K77 deacetylation tended to interplay with HSP40 and CHIP for protein degradation (Seo et al., 2016). Furthermore, deacetylated K77 would weaken HSP70 ATP hydrolysis and ATP binding ability, but the deacetylated K126 could enhance HSP70 ATP binding (Seo et al., 2016; Sun et al., 2019). Hence, in the subsequent study, K77 will be the superior target to unravel the role of HSC70-4 in BmNPV invasion. Also, K246 acetylation of HSC70-4 would still be our research goal for future baculovirus analysis about autophagy, and in a recent study, we reported that the autophagy-related gene 8 (*Atg8*) acetylation triggered by BmNPV regulates autophagy initiation (Xue et al., 2019).

Recent studies reported that the lysine acetylation could compete with ubiquitination to stabilize the protein (Ma et al., 2020). In the current study, different from VER-induced HSC70-4 degradation upon BmNPV stimulus, the deacetylation-mimic K77R blocked the ubiquitination of lysine, which might contribute to avoiding its degradation under normal circumstances (Figure 4A), which may be associated with allosteric conformational change failure of the ATP/ADP binding cycle (Seo et al., 2016). According to a previous study, HSC70-4 accumulated in the nucleus at the late infectious stage (Iwanaga et al., 2014). Similarly, the K77 deacetylation had a vital role in this nuclear import during BmNPV infection (Figure 4B), which might be associated with increased genome replication.

To detect whether the K77 acetylation affects the interacting partner of HSC70-4, we applied the yeast two-hybrid assay (Y2H). These results were consistent with those of a previous study that K77 acetylated HSP70 completely blocked its interaction with CHIP without any protein sequence mutation (Seo et al., 2016), and the consensus between HSP70 and HSC70 may provide novel insights into the categorization of these analogous molecules. HSC70-4, HSC70-3, HSC70-5, and HSC70-2 in *Bombyx mori* are constitutively expressed HSP70. HSC70-2 and HSC70-4 were located in the cytoplasm; HSC70-3 was in the endoplasmic reticulum. HSC70-5 was expressed in the mitochondria (Wang et al., 2012). Different cellular localization of HSP70 possibly decides the functional variety. A previous study showed differential effects of HSC70 and HSP70 on the intracellular trafficking and functional expression of epithelial sodium channels (Goldfarb et al., 2006), while the difference between HSC70 and HSP70 in baculovirus infection needs to be elucidated further.

In several investigations, the HSP/HSC70 colocalized with ubiquitinated proteins during the baculovirus infection (Lyupina et al., 2011, 2013; Guo et al., 2015). Linked to K77 deacetylation and interaction with E3 ubiquitin ligase CHIP, we found that the ubiquitin-proteasome system might contribute to the HSC70-4 nuclear import during the infectious process. A recent study also found that the ubiquitin-proteasome system is crucial for BmNPV polyhedrin expression and BV production (Katsuma et al., 2011). In the present study, the ubiquitin-proteasome is also required for viral genome replication (Figure 6A) and proliferation (Figure 6B) and can compromise K77 deacetylation-mediated HSC70-4 nuclear import (Figure 6C); also, the number of genome copies increase (Figure 6E) after BmNPV challenge. This phenomenon might imply that HSC70-4 nuclear accumulation is dependent on the ubiquitin-proteasome system for facilitating BmNPV replication.

## Data Availability Statement

The original contributions presented in the study are included in the article/Supplementary Material, further inquiries can be directed to the corresponding author.

## Author Contributions

FM, XC, and JN investigated the experiments, interpreted the data, and drafted the manuscript. YZ, JL, and XG provided critical data analysis and technical support. MM, YQ, and WY supervised the study. All authors reviewed the manuscript.

## Conflict of Interest

The authors declare that the research was conducted in the absence of any commercial or financial relationships that could be construed as a potential conflict of interest.

## Supplementary Material

Supplementary Figure 1 Kac sites of HSC70-4 response upon BmNPV stimulation. **(A)** The miniature architecture of HSC70-4 functional domains, including nucleotide-binding domain (blue section, 1–382 aa), substrate-binding domain (green section, 383–537 aa), and C-terminal domain (yellow section, 538–649 aa), was determined with a myriad of acetylated lysine sites in disparate segments (Kundrat and Regan, 2010; Muller et al., 2013; Gao et al., 2015; Verdin and Ott, 2015; Seo et al., 2016), and lysine sites in the red bar were identified in our previous profile. **(B)** Five Kac sites of HSC70-4 upon BmNPV trigger were identified by nano-HPLC/MS/MS.

Supplementary Figure 2 K77 acetylation affects the interaction between HSC70-4 and HOP. Yeast two-hybrid assay of the interaction between HSC70-4/K77Q/K77R and *B. mori* HOP. pGBKT7-HSC70-4/K77Q/K77R and pGADT7-HOP is the experimental group; pGBKT7-53 and pGADT7-T are positive control; pGBKT7-Lam and pGADT7-T, pGBKT7 and pGADT7-HOP, pGBKT7-HSC70-4/K77Q/K77R, and pGADT7 are regarded as negative controls.

Supplementary Figure 3 Stability of HSC70-4 upon BmNPV challenge. BmN cellular endogenous HSC70-4 stability dynamics after BmNPV infection were assessed at several different time points by immunoblotting assay. The viral structure protein Gp64 represented the BmNPV infectious process successfully. β-tubulin is used as loading control.

Supplementary Table 1 The primers involved in this study.

## References

[B1] Breitenbach J. E. Popham H. J. R. (2013). Baculovirus replication induces the expression of heat shock proteins *in vivo* and *in vitro*. *Arch Virol.* 158 1517–1522. 10.1007/s00705-013-1640-8 23443933

[B2] Fernández-Fernández M. R. Gragera M. Ochoa-Ibarrola L. Quintana-Gallardo L. Valpuesta J. M. (2017). Hsp70-a master regulator in protein degradation. *FEBS Lett.* 591 2648–2660. 10.1002/1873-3468.12751 28696498

[B3] Fujimoto H. Higuchi M. Koike M. Ode H. Pinak M. Bunta J. K. (2012). possible overestimation of the effect of acetylation on lysine residues in KQ mutant analysis. *J. Comput. Chem.* 33 239–246. 10.1002/jcc.21956 22072565

[B4] Gao W. Xiao R. Peng B. Xu H. Shen H. Huang M. (2015). Arginine methylation of HSP70 regulates retinoid acid-mediated RARβ2 gene activation. *Proc. Natl. Acad. Sci. U.S.A.* 122 E3327–E3336. 10.1073/pnas.1509658112 26080448 PMC4491752

[B5] Gething M. J. Sambrook J. (1992). Protein folding in the cell. *Nature* 355 33–45. 10.1038/355033a0 1731198

[B6] Goldfarb S. B. Kashlan O. B. Watkins J. N. Suaud L. Yan W. Kleyman T. R. (2006). Differential effects of Hsc70 and Hsp70 on the intracellular trafficking and functional expression of epithelial sodium channels. *Proc. Natl. Acad. Sci. U.S.A.* 103 5817–5822. 10.1073/pnas.0507903103 16585520 PMC1458656

[B7] Guo Z. J. Tao L. X. Dong X. Y. Yu M. H. Tian T. Tang X. D. (2015). Characterization of aggregate/aggresome structures formed by polyhedrin of *Bombyx mori* nucleopolyhedrovirus. *Sci. Rep.* 5:14601. 10.1038/srep14601 26440217 PMC4594129

[B8] Ho S. N. Hunt H. D. Horton R. M. Pullen J. K. Pease L. R. (1989). Site-directed mutagenesis by overlap extension using the polymerase chain reaction. *Gene* 77 51–59. 10.1016/0378-1119(89)90358-2 2744487

[B9] Hu D. Xue S. Zhao C. Wei M. Yan H. Quan Y. (2018). Comprehensive profiling of lysine acetylome in baculovirus infected silkworm (*Bombyx mori*) cells. *Proteomics* 18:201700133. 10.1002/pmic.201700133 29150924

[B10] Huang R. Xu Y. Wan W. Shou X. Qian J. You Z. (2015). Deacetylation of nuclear LC3 drives autophagy initiation under starvation. *Mol. Cell* 57 456–466. 10.1016/j.molcel.2014.12.013 25601754

[B11] Iwanaga M. Shibano Y. Ohsawa T. Fujita T. Katsuma S. Kawasaki H. (2014). Involvement of HSC70-4 and other inducible HSPs in *Bombyx mori* nucleopolyhedrovirus infection. *Virus Res.* 179 113–118. 10.1016/j.virusres.2013.10.028 24211667

[B12] Jiang L. Goldsmith M. R. Xia Q. (2021a). Advances in the arms race between silkworm and baculovirus. *Front. Immunol.* 12. 10.3389/fimmu.2021.628151 PMC7900435 33633750

[B13] Jiang L. Xia Q. (2014). The progress and future of enhancing antiviral capacity by transgenic technology in the silkworm *Bombyx mori*. *Insect. Biochem. Mol. Biol.* 48 1–7. 10.1016/j.ibmb.2014.02.003 24561307

[B14] Jiang L. Xie E. Guo H. Sun Q. Liuli H. Wang Y. (2021b). Heat shock protein 19.9 (Hsp19.9) from *Bombyx mori* is involved in host protection against viral infection. *Dev. Comp. Immunol.* 114:103790. 10.1016/j.dci.2020.103790 32784012

[B15] Katsuma S. Tsuchida A. Matsuda-Imai N. Kang W. Shimada T. (2011). Role of the ubiquitin-proteasome system in *Bombyx mori* nucleopolyhedrovirus infection. *J. Gen. Virol.* 92 699–705. 10.1099/vir.0.027573-0 21084493

[B16] Kundrat L. Regan L. (2010). Identification of residues on Hsp70 and Hsp90 ubiquitinated by the cochaperone CHIP. *J. Mol. Biol.* 395 587–594. 10.1016/j.jmb.2009.11.017 19913553 PMC2917188

[B17] Lindquist S. Craig E. A. (1988). The heat-shock proteins. *Annu. Rev. Genet.* 22 631–677. 10.1146/annurev.ge.22.120188.003215 2853609

[B18] Liu X. Chen K. Cai K. Yao Q. (2008). Determination of protein composition and host-derived proteins of *Bombyx mori* nucleopolyhedrovirus by 2-dimensional electrophoresis and mass spectrometry. *Intervirology* 51 369–376. 10.1159/000193462 19151556

[B19] Lyupina Y. V. Abaturova S. B. Erokhov P. A. Orlova O. V. Beljelarskaya S. N. Mikhailov V. S. (2013). Proteotoxic stress induced by *Autographa californica* nucleopolyhedrovirus infection of *Spodoptera frugiperda* Sf9 cells. *Virology* 436 49–58. 10.1016/j.virol.2012.10.018 23123012

[B20] Lyupina Y. V. Dmitrieva S. B. Timokhova A. V. Beljelarskaya S. N. Zatsepina O. G. Evgen’ev M. B. (2010). An important role of the heat shock response in infected cells for replication of baculoviruses. *Virology* 406 336–341. 10.1016/j.virol.2010.07.039 20708767

[B21] Lyupina Y. V. Orlova O. V. Abaturova S. B. Beljelskaya S. N. Lavrov A. N. Mikhailov V. S. (2014). Egress of budded virions of *Autographa californica* nucleopolyhedrovirus does not require activity of *Spodoptera frugiperda* HSP/HSC70 chaperones. *Virus Res.* 192 1–5. 10.1016/j.virusres.2014.08.002 25128466

[B22] Lyupina Y. V. Zatsepina O. G. Timokhova A. V. Orlova O. V. Kostyuchenko M. V. Beljelarskaya S. N. (2011). New insights into the induction of the heat shock proteins in baculovirus infected insect cells. *Virology* 421 34–41. 10.1016/j.virol.2011.09.010 21982219

[B23] Ma Y. Wu C. Liu J. Liu Y. Lv J. Sun Z. (2020). The stability and antiapoptotic activity of Bm30K-3 can be improved by lysine acetylation in the silkworm, *Bombyx mori*. *Arch. Insect. Biochem. Physiol.* 103:e21649. 10.1002/arch.21649 31777104

[B24] Mao F. Lei J. Obeng E. Wei M. Zhao C. Quan Y. (2018). Quantitative proteomics of *Bombyx mori* after BmNPV challenge. *J. Proteomics* 181 142–151. 10.1016/j.jprot.2018.04.010 29674014

[B25] Mao F. Zhu Y. Gao X. Chen X. Ngowo J. Miao M. (2020). HSP/HSC70 activity is required for *Bombyx mori* nucleopolyhedrovirus replication at the early infectious phase. *Microb. Pathog.* 153:104647. 10.1016/j.micpath.2020.104647 33246089

[B26] Mawatari T. Ninomiya I. Inokuchi M. Harada S. Hayashi H. Oyama K. (2015). Valproic acid inhibits proliferation of HER2-expressing breast cancer cells by inducing cell cycle arrest and apoptosis through Hsp70 acetylation. *Int. J. Oncol.* 47 2073–2081. 10.3892/ijo.2015.3213 26497673 PMC4665753

[B27] Muller P. Ruckova E. Halada P. Coates P. J. Hrstka R. Lane D. P. (2013). C-terminal phosphorylation of Hsp70 and Hsp90 regulates alternate binding to co-chaperones CHIP and HOP to determine cellular protein folding/degradation balances. *Oncogene* 32 3101–3110. 10.1038/onc.2012.314 22824801

[B28] Ohsawa T. Fujimoto S. Tsunakawa A. Shibano Y. Kawasaki H. Iwanaga M. (2016). Cloning and characterization of carboxyl terminus of heat shock cognate 70-interacting protein gene from the silkworm, *Bombyx mori*. *Comp. Biochem. Physiol. B Biochem. Mol. Biol.* 201 29–36. 10.1016/j.cbpb.2016.06.009 27378406

[B29] Park Y. H. Seo J. H. Park J. H. Lee H. S. Kim K. W. (2017). Hsp70 acetylation prevents caspase-dependent/independent apoptosis and autophagic cell death in cancer cells. *Int. J. Oncol.* 51 573–578. 10.3892/ijo.2017.4039 28627586

[B30] Seo J. H. Park J. H. Lee E. J. Vo T. T. L. Choi H. Kim J. Y. (2016). ARD1-mediated Hsp70 acetylation balances stress-induced protein refolding and degradation. *Nat. Commun.* 7:12882. 10.1038/ncomms12882 27708256 PMC5059642

[B31] Shang Q. Wu P. Huang H. L. Zhang S. L. Tang X. D. Guo X. J. (2020). Inhibition of heat shock protein 90 suppresses *Bombyx mori* nucleopolyhedrovirus replication in *B. mori*. *Insect. Mol. Biol.* 29 205–213. 10.1111/imb.12625 31621968

[B32] Shen Y. Feng M. Wu X. (2018). *Bombyx mori* nucleopolyhedrovirus ORF40 is essential for budded virus production and occlusion-derived virus envelopment. *J. Gen. Virol.* 99 837–850. 10.1099/jgv.0.001066 29676725

[B33] Sun F. Jiang X. Wang X. Bao Y. Feng G. Liu H. (2019). Vincristine ablation of Sirt2 induces cell apoptosis and mitophagy via Hsp70 acetylation in MDA-MB-231 cells. *Biochem. Pharmacol.* 162 142–153. 10.1016/j.bcp.2018.10.021 30352233

[B34] Surtees R. Dowall S. D. Shaw A. Armstrong S. Hewson R. Carroll M. W. (2016). Heat shock protein 70 family members interact with Crimean-Congo hemorrhagic fever virus and Hazara virus nucleocapsid proteins and perform a functional role in the Nairovirus replication cycle. *J. Virol.* 90 9305–9316. 10.1128/JVI.00661-16 27512070 PMC5044845

[B35] Taguwa S. Maringer K. Li X. Bernal-Rubio D. Rauch J. N. Gestwicki J. E. (2015). Defining Hsp70 subnetworks in Dengue virus replication reveals key vulnerability in flavivirus infection. *Cell* 163 1108–1123. 10.1016/j.cell.2015.10.046 26582131 PMC4869517

[B36] Taguwa S. Yeh M. T. Rainbolt T. K. Nayak A. Shao H. Gestwicki J. E. (2019). Zika virus dependence on host Hsp70 provides a protective strategy against infection and disease. *Cell Rep.* 26 906–920. 10.1016/j.celrep.2018.12.095 30673613 PMC6709865

[B37] Tang S. Zhao Q. Yi Y. Zhang Z. Li Y. (2005). Homologous region 3 from *Bombyx mori* nucleopolyhedrovirus enhancing the transcriptional activity of heat shock cognate 70-4 promoter from *Bombyx mori* and *Bombyx mandarian* in vitro and in vivo. *Biosci. Biotechnol. Biochem.* 69 1014–1017. 10.1271/bbb.69.1014 15914923

[B38] Verdin E. Ott M. (2015). 50 years of protein acetylation: from gene regulation to epigenetics, metabolism and beyond. *Nat. Rev. Mol. Cell Biol.* 16 258–264. 10.1038/nrm3931 25549891

[B39] Wang L. L. Lin H. J. Wang Y. Li Z. Zhou Z. Y. (2012). Chromosomal localization and expressional profile of heat shock protein 70 family genes in silkworm, *Bombyx mori*. *Sci. Sericul.* 38 617–623. 10.13441/j.cnki.cykx.2012.04.010

[B40] Wu M. Y. Fu J. Xiao X. Wu J. Wu R. C. (2014). MiR-34a regulates therapy resistance by targeting HDAC1 and HDAC7 in breast cancer. *Cancer Lett.* 354 311–319. 10.1016/j.canlet.2014.08.031 25173798

[B41] Xia Q. Zhou C. Lu C. Cheng D. Dai F. Li B. (2004). A draft sequence for the genome of the domesticated silkworm (*Bombyx mori*). *Science* 306 1937–1940. 10.1126/science.1102210 15591204

[B42] Xue S. Mao F. Hu D. Yan H. Lei J. Obeng E. (2019). Acetylation of BmAtg8 inhibits starvation-induced autophagy initiation. *Mol. Cell Biochem.* 457 73–81. 10.1007/s11010-019-03513-y 30877510

[B43] Yang F. Zhu B. Liu J. Liu Y. Jiang C. Sheng Q. (2020). The effect of acetylation on the protein stability of BmApoLp-III in the silkworm, *Bombyx mori*. *Insect. Mol. Biol.* 29 104–111. 10.1111/imb.12613 31390480

[B44] Yang Y. Fiskus W. Yong B. Atadja P. Takahashi Y. Pandita T. K. (2013). Acetylation hsp70 and KAP1-mediated Vps34 SUMOylation is required for autophagosome creation in autophagy. *Proc. Natl. Acad. Sci. U.S.A.* 110 6841–6846. 10.1073/pnas.1217692110 23569248 PMC3637746

[B45] Yu W. Du C. Y. Quan Y. P. Nie Z. M. Chen J. Lv Z. B. (2013a). Characterization of late gene expression factor LEF-10 from *Bombyx mori* nucleopolyhedrovirus. *Virus Res.* 175 45–51. 10.1016/j.virusres.2013.03.022 23603137

[B46] Yu W. Li Q. Yao Y. Quan Y. Zhang Y. (2013b). Two novel 30K proteins overexpressed in baculovirus system and their antiapoptotic effect in insect and mammalian cells. *Int. J. Genomics* 2013:323592. 10.1155/2013/323592 23862133 PMC3686079

[B47] Zhao C. Zhang C. Chen B. Shi Y. Quan Y. Nie Z. (2016). A DNA binding protein is required for viral replication and transcription in *Bombyx mori* nucleopolyhedrovirus. *PLoS One* 11:e0159149. 10.1371/journal.pone.0159149 27414795 PMC4945074

[B48] Zhou Y. Wu C. Sheng Q. Jiang C. Chen Q. Lv Z. (2016). Lysine acetylation stabilizes SP2 protein in the silkworm *Bombyx mori*. *J. Insect. Physiol.* 9 56–62. 10.1016/j.jinsphys.2016.06.008 27374983

