# Supplementary material for: Deacetylation of HSC70-4 Promotes Bombyx mori Nucleopolyhedrovirus Proliferation via Proteasome-Mediated Nuclear Import
Source: Front Physiol. 2021 Feb 19;12:609674. doi: 10.3389/fphys.2021.609674 (PMC7935516; doi:10.3389/fphys.2021.609674)
Supplement: Supplementary Figure 1 — Kac sites of HSC70-4 response upon BmNPV stimulation. (A) The miniature architecture of HSC70-4 functional domains, including nucleotide-binding domain (blue section, 1–382 aa), substrate-binding domain (green section, 383–537 aa), and C-terminal domain (yellow section, 538–649 aa), was determined with a myriad of acetylated lysine sites in disparate segments (Kundrat and Regan, 2010; Muller et al., 2013; Gao et al., 2015; Verdin and Ott, 2015; Seo et al., 2016), and lysine sites in the red bar were identified in our previous profile. (B) Five Kac sites of HSC70-4 upon BmNPV trigger were identified by nano-HPLC/MS/MS. [file Data_Sheet_1.docx]

**Table S1 Primers involved in this study**

| Primers | Sequences (5’-3’) |
| --- | --- |
| HSC70-4-898-EcoRI-F | TATTGAATTCGCTCGCTTCGAGGAGCTGAACGCC |
| HSC70-4-1801-NotI-R | TTATGCGGCCGCTTCCAATTCTTTCTGCTTGTGCTCTAG |
| HSC70-4-PstI-F  HSC70-4-XhoI-R  K71-F (K/Q, K/R)  K71-R (K/Q, K/R)  K77-F (K/Q, K/R)  K77-R (K/Q, K/R)  K88-F (K/Q, K/R)  K88-R (K/Q, K/R)  K126-F (K/Q, K/R)  K126-R (K/Q, K/R)  K246-F (K/Q, K/R)  K246-R (K/Q, K/R)  K524-F (K/Q, K/R)  K524-R (K/Q, K/R)  EGFP/mCherry-PstI-F  EGFP/mCherry-6×Glycine-R  HSC70-4-6×Glycine-F  mCherry-NotI-F  HOP-6×Glycine-F  CHIP-6×Glycine-F  HOP-XhoI-R  CHIP-XhoI-R | AACTGCAGATGGCAAAAGCACCCGC  CCGCTCGAGATCGACCTCCTCGATGGTGGG  TTCGATGCC**CAA**CGTCTCATC **/** TTCGATGCC**AGA**CGTCTCATC  GATGAGACG**TTG**GGCATCGAA **/** GATGAGACG**TCT**GGCATCGAA  ATCGGACGT**CAG**TTCGAAGA **/** ATCGGACGT**AGG**TTCGAAGA  TCTTCGAA**CTG**ACGTCCGAT **/** TCTTCGAA**CCT**ACGTCCGAT  GCCGACATG**CAG**CACTGGCCT **/** GCCGACATG**AGG**CACTGGCCT  AGGCCAGTG**CTG**CATGTCGGC **/** AGGCCAGTG**CCT**CATGTCGGC  GTGCTTACG**CAA**ATGAAGGAA **/** GTGCTTACG**AGA**TGAAGGAA  TTCCTTCAT**TTG**CGTAAGCAC **/** TTCCTTCAT**TCT**CGTAAGCAC  CAGGAGTTC**CAG**AGGAAATAC **/** CAGGAGTTC**AGG**AGGAAATAC  GTATTTCCT**CTG**GAACTCCT **/** GTATTTCCT**CCT**GAACTCCT  TAATGAGGCAGAG**CAG**TACA **/** TAATGAGGCAGAG**AGG**TACA  TGTA**CTG**CTCTGCCTCATTA **/** TGTA**CCT**CTCTGCCTCATTA  AACTGCAGATGGTGAGCAAGGGC  ACCTCCCCCGCCACCTCCCTTGTACAGCTCGTCC  GGAGGTGGCGGGGGAGGTATGGCAAAAGCACCCGC  ATTTGCGGCCGCATGTTGAGCAAGG  GGAGGTGGCGGGGGAGGTATGGATAAGGTGGAACAGTT  GGAGGTGGCGGGGGAGGTATGAGCAAACATATG  CCGCTCGAGATGTAGGGCGATGAGGCC  CCGCTCGAGATAGTCGAGGGCCCA |

Footnotes: Underlined bases represent the restriction enzyme; Black bold bases indicate original lysine residue; Red bold letters mean acetylation-mimic glutamine residue; Green bold letters mean deacetylation-mimic arginine residue; Double underlined bases represent 6×glycine linker polypeptide bases.


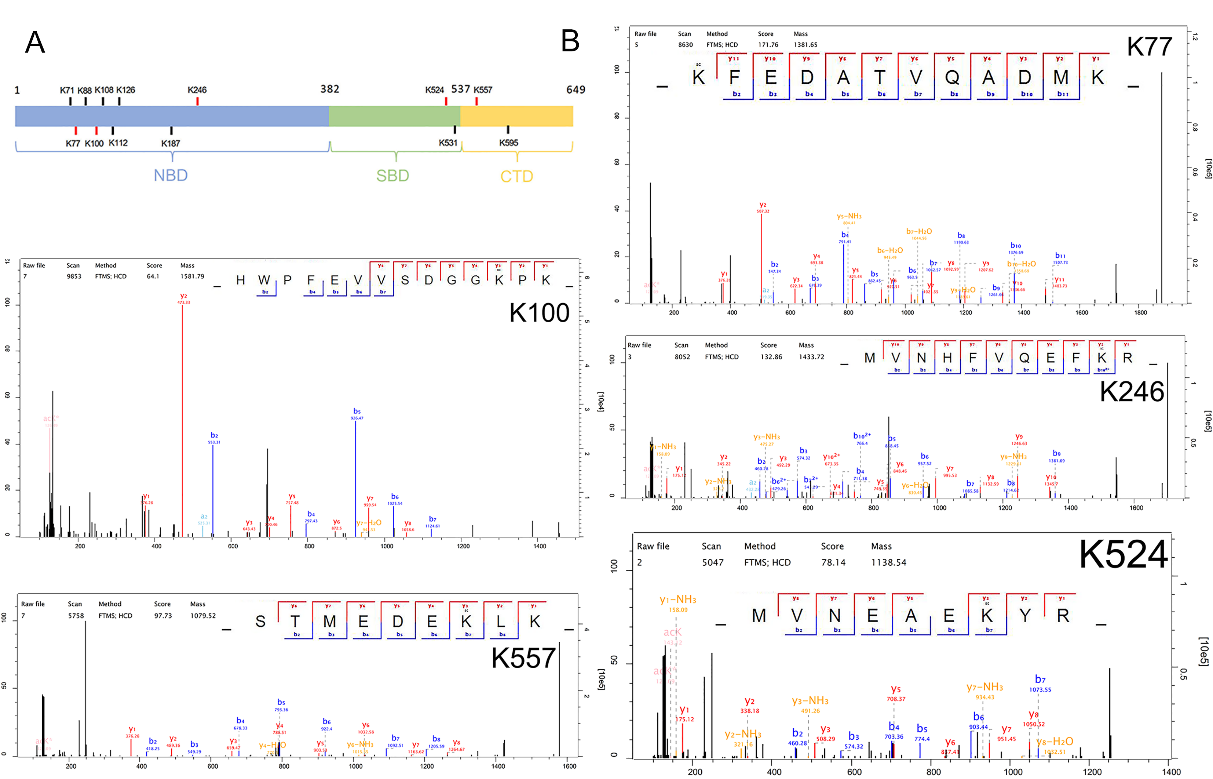


**Fig. S1**

**
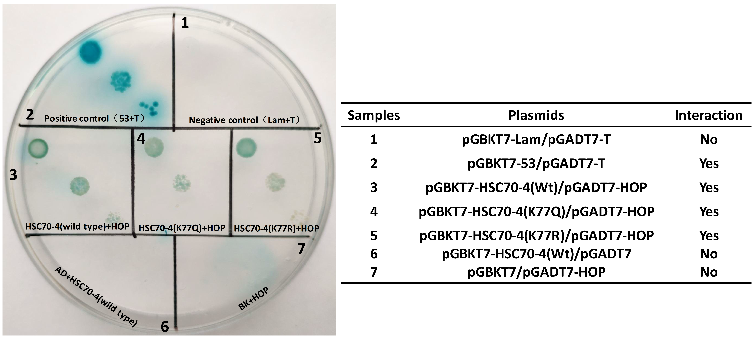
**

**Fig. S2**


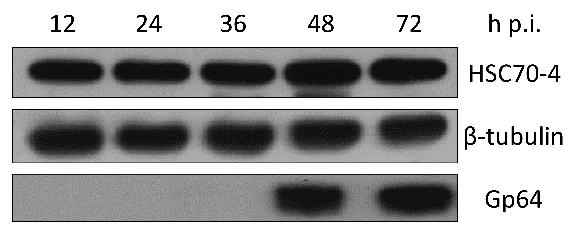


**Fig. S3**

Stability of HSC70-4 upon BmNPV challenge. BmN cellular endogenous HSC70-4 stability dynamics after BmNPV infection were assessed at several different time points by immunoblotting assay. The viral structure protein Gp64 represented the BmNPV infectious process successfully. β-tubulin is used as loading control.
